# An Efficient Polymer Inclusion Membrane-Based Device for Cd Monitoring in Seawater

**DOI:** 10.3390/membranes8030061

**Published:** 2018-08-10

**Authors:** Ibrahim Ait Khaldoun, Lynda Mitiche, Amar Sahmoune, Clàudia Fontàs

**Affiliations:** 1 Department of Chemistry, University of Girona, C/Maria Aurèlia Capmany 69, 17003 Girona, Spain; iaitkhaldoun@yahoo.fr; 2 Equipe de Recherche Matériaux et Procédés pour l’Environnement, Université Mouloud Mammeri Tizi-Ouzou, Tizi-Ouzou 15000, Algeria; lynda76dz@yahoo.fr (L.M.); asahmoune@yahoo.fr (A.S.)

**Keywords:** cadmium, polymer inclusion membrane, trihexyl (tetradecyl) phosphonium chloride (THTDPCl), seawater

## Abstract

A novel and simple device that includes a polymer inclusion membrane (PIM) has been prepared and tested for the first time to detect low concentration levels of cadmium in seawater. The ionic liquid trihexyl (tetradecyl) phosphonium chloride (THTDPCl) has been shown to be an effective extractant when incorporated in a PIM that uses cellulose triacetate (CTA) as a polymer. However, it has been reported that the use of a plasticizer is mandatory to ensure an effective transport, which uses both ultrapure water and a nitric acid solution as a stripping phase. A special device incorporating a PIM made of 50% CTA, 40% nitrophenyl octyl ether (as a plasticizer), and 10% THTDPCl, effectively allows the quantitative transport and preconcentration of 10 µg L^−1^ Cd from seawater samples to a stripping phase consisting of 0.5 M HNO_3_ solution. This study shows that the efficiency of the PIM system is not affected by high salinity nor the presence of large amounts of other ions, and can thus facilitate Cd monitoring in seawater samples.

## 1. Introduction

The release of different pollutants into the environment, which has increased noticeably as a result of industrialization, is having an alarming effect on its quality. Heavy metals are the most important of these pollutants due to their non-biodegradability. In the case of cadmium, it is toxic even at trace concentrations. However, what is of most concern about this metal is its capacity for bioaccumulation. Cd in water is due nearly exclusively to industrial discharges (e.g., from electroplating, paint-making, the manufacture of plastics, etc.) and landfill leachates. High metal accumulation by organisms, and their consumption in the food chain, is a cause of major health problems and it is known that the marine food chain is seriously affected by Cd in seawater [1]. As a result, the accurate determination of this metal in seawater systems is receiving significant attention.

The presence of trace amounts of heavy metals in environmental samples is usually determined by either electrochemical or spectrophotometric techniques. However, the direct analysis of metals in seawater presents some difficulties due to the high salt content and low metal concentration, which results in matrix interference and insufficient precision, and so sample preparation is normally necessary prior to analysis. A possible solution to isolate Cd from seawater samples is to use an extractant diluted in an organic solvent. Taking advantage of the fact that Cd is mainly present as an anionic chlorocomplex in seawater, typical extractants used for its extraction in this media are anion exchangers, such as the quaternary ammonium salt Aliquat 336, which is an ionic liquid (IL) at room temperature. This IL, dissolved in kerosene with 0.25 M dodecan-1-ol, formed a liquid membrane which was used to impregnate the pores of a fiber to act as a three phase solvent bar micro-extraction system [2]. The impregnated fiber was effective to preconcentrate Cd from seawater samples into a 1.5 M HNO_3_ solution (used as a stripping phase). More recently, a supported liquid membrane (SLM) containing this IL was developed and studied in both flat-sheet and hollow fiber configurations to separate Cd from complex aqueous samples [3]. It was found that using a 2 M HCl solution containing Cd, Cu, Ni, and Pb as a feed phase, only Cd was transported. Additionally, Cd could be preconcentrated from spiked seawater samples using the SLM in hollow fiber configurations. Further, other IL based on phosphonium derivatives have also been proved to be effective extractants for Cd in chloride media. Singh et al. used Cyphos IL 102, diluted in toluene (trihexyl (tetradecyl) phosphonium bromid), to extract Cd and Zn from a hydrochloric acid medium [4]. Cyphos IL 104 (trihexyl (tetradecyl) phosphonium bis (2,4,4-trimethylpentyl) phosphinate), incorporated in a polymeric matrix, was shown to effectively remove Cd (II) from both NaCl and HCl solutions into a 1 M H_2_SO_4_ solution, which acted as a receiving phase [5]. Cyphos IL 101 (trihexyl (tetradecyl) phosphonium chloride) was immobilized in a synthetic resin (Amberlite XAD-7) to prepare a solvent impregnated resin (SIR). This SIR effectively allowed Cd (II) sorption in HCl solutions, and the extraction was found to occur via an exchange mechanism involving the reaction between chloroanionic Cd species and the phosphonium cation (R_3_R′ P^+^) [6].

Polymer inclusion membranes (PIMs), which are advanced liquid extracting membranes, can also be used to entrap the extractants. These membranes form homogeneous and non-porous films that have shown to be more stable than SLMs. PIMs are composed of a base polymer, providing mechanical strength, an extractant, which is immobilized within the chains of the base polymer, and in some cases, a plasticizer, which provides elasticity to the membrane. PIMs are of great interest for analytical purposes since they are very versatile and can be used for sample separation, sample preconcentration, and passive sampling, among others [7]. The use of devices incorporating PIMs allow compounds to be easily preconcentrated by using smaller volumes of the receiving phase, in comparison to the source solution. A PIM-device, containing Aliquat 336 as the carrier, allowed for the detection of As in groundwater [8]. A similar PIM-device was designed for the monitoring of antibiotic [9] Zn [10] or ammonia [11] in natural waters. However, to the best of our knowledge, there is no previous work on the use of such a PIM-device for the preconcentration of metals from seawater samples. Taking the complexity of natural water into account, it is important to investigate whether the high salinity or the presence of large amounts of other ions can affect the performance of the PIM-device.

In this study, we have developed a PIM incorporating IL trihexyl (tetradecyl) phosphonium chloride (THTDPCl) for the transport of Cd ions in a high chloride medium and have presented the results obtained using a PIM-device for the preconcentration of Cd from seawater samples.

## 2. Materials and Methods

### 2.1. Chemicals

Aqueous solutions of Cd were prepared by the dilution of the corresponding stock solution (1000 mgL^−1^, Romil, Cambridge, UK) and by the addition of NaCl (Panreac, Barcelona, Spain) to reach the desired chloride concentration. Nitric acid (Fluka, Buchs, Switzerland) and EDTA (Panreac, Spain) were used to prepare the stripping solutions and consisted of 0.5 M HNO_3_ or 0.1M EDTA. All chemicals were of analytical reagent grade and the solutions were prepared with ultrapure water obtained by purification through a Milli-Q Plus system (Millipore Iberica SA, Madrid, Spain).

The extractant—trihexyl (tetradecyl) phosphonium chloride (THTDPCl)—was purchased from Aldrich (Germany). The polymer cellulose triacetate (CTA) and the plasticizers 2-nitrophenyl octyl ether (NPOE), 2-Fluorophenyl 2-nitrophenyl ether (FPNPOE), and dibutyl sebacate (DBS), were purchased from Fluka Chemie (Switzerland), while chloroform was from Panreac (Spain). All of these reagents were used as received.

Spiked seawater was obtained after adding the appropriate amount of Cd stock solution to seawater collected from the Mediterranean, it had the following chemical characteristics, pH 8.2, conductivity 62 mS, 0.62 M Cl^−^, 0.68 M Na^+^, 4325 mg L^−1^ SO_4_^2−^, 1415 mg L^−1^ Mg^2+^, Ca^2+^, K^+^, HCO_3_^−^, and the concentration was <1000 mg L^−1^.

### 2.2. Polymer Inclusion Membranes Preparation and Stability Test

PIMs were prepared by following the procedure described by Garcia-Rodríguez [12]. The CTA amount was fixed at 200 mg and other components were added to achieve the desired concentration of plasticizer and carrier. The composition of the PIMs (quoted in mass percentage) tested in this study, as well as the plasticizers characteristics, can be seen in Table 1.

The stability of PIM 2 was investigated in terms of mass loss, which is related to the loss of the carrier [13]. For that, PIM pieces of approximately 2 cm × 2 cm were dipped in 25 mL of ultrapure water and were agitated in an orbital mixer for 24 h. Membranes were weighed before and after this procedure, and the mass loss was calculated.

### 2.3. Instrumentation

Cd determination in the feed and stripping phases was made by atomic emission spectrometry with an ICP-AES instrument (Varian Liberty RL, Victoria, Australia), and a KS250 multiple stirrer (Ika, Labortechnik, Staufen, Germany) was used for transport experiments.

The scanning electron microscopy (SEM) observations of the membrane samples were made using a FE-SEM Hitachi S-4100 (Tokyo, Japan). The samples were placed on a stub and coated with carbon (model K950 turbo evaporator, Emitech, Lohmar, Germany). Digital images were collected and processed by Quarz PCI program (Vancouver, BC, Canada).

### 2.4. Transport and Preconcentration Experiments

To evaluate the use of PIMs for the transport of Cd, some experiments were performed in a two compartment membrane cell, described elsewhere [14], with 190 mL of both feed solution (10 mg L^−1^ Cd in 2 M NaCl) and a stripping phase was used that incorporated a PIM of 11 cm^2^ in area. Preconcentration studies were done using a device containing a PIM of 2.5 cm^2^ in contact with 100 mL of feed solution in agitation. In order to allow for preconcentration, only 5 mL of a stagnant stripping solution was used. A scheme of the PIM-device and the whole setup can be found in [15]. Samples were withdrawn and analysed by ICP-AES. The percentage of Cd extraction, *E*(%), was then calculated by Equation (1):

(1) $$E(\%)=\frac{{\left[Cd\right]}_{feed_{\left(0\right)}}-{\left[Cd\right]}_{feed_{\left(t\right)}}}{{\left[Cd\right]}_{feed_{\left(0\right)}}}\times 100$$

where [*Cd*]*_feed_*_(0)_ is the initial Cd concentration in the water sample, whereas [*Cd*]*_feed_*_(*t*)_ is the metal concentration in the source solution at the end of the experiment (24 h). Cd transport efficiency (*TE*) was determined using Equation (2):

(2) $$TE(\%)=\frac{{\left[Cd\right]}_{strip_{\left(t\right)}}}{{\left[Cd\right]}_{feed_{\left(0\right)}}}\;\frac{1}{Vr}\times 100$$

where [*Cd*]*_strip_*_(*t*)_ denotes the metal concentration in the stripping compartment at the end of the contact time. The volume ratio between feed solution and stripping solutions is denoted by *Vr*. For the two-compartment cell *Vr* = 1, and for the PIM device *Vr* = 20.

## 3. Results

### 3.1. Characteristics of the Polymer Inclusion Membranes (PIMs)

**Figure 1:**
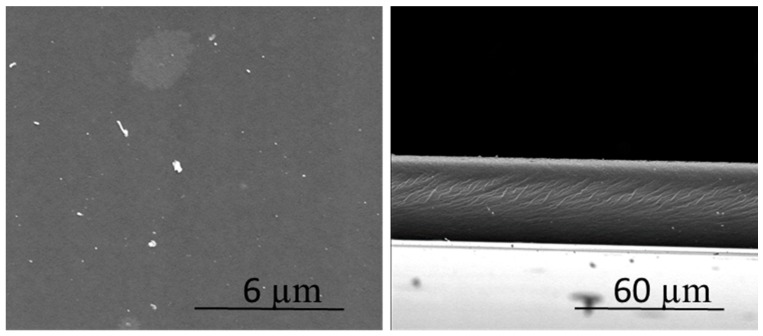
SEM images of a PIM with a composition of 50% CTA, 30% THTDPCl, and 20% NPOE. (**Left**) surface; (**right**) cross-section.

All the PIMs investigated in this study were effectively prepared indistinctly when they only contained CTA and the extractant THTDPCl or when they incorporated the added plasticizers NPOE, FNPOE, and DBS. The addition of a plasticizer reduces the glass transition temperature of the polymer and can also facilitate the transport of the target species through the PIM [16]. All the membranes were transparent, homogeneous, and mechanically stable. Figure 1 shows the morphology of the PIM containing NPOE as a plasticizer (M2), and it can be seen that the resulting membrane was dense and without pores.

The stability of the membrane M2 was investigated in terms of the extent of leaching of the IL from the membrane in aqueous solutions. Membrane mass loss was found to be 9 ± 1% (*n* = 3). This value is much lower than the obtained value for a PIM made of polyvinyl chloride (PVC), and the similar content of Aliquat 336, which was reported to be 33% [17].

### 3.2. Transport Experiments

**Figure 2:**
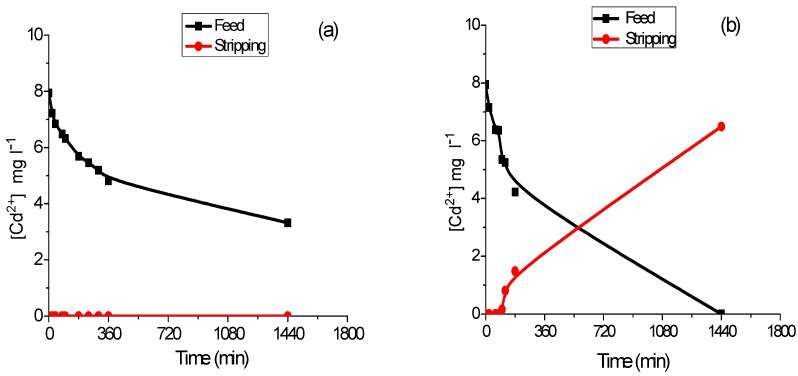
Transient concentration curves in Cd (II) transport experiments involving PIM 1 (**a**) and PIM 2 (**b**).

All the produced membranes (M1–M4) were used in the investigation of the transport of Cd from a 2 M NaCl solution to an ultra-pure water-stripping phase. Transport efficiency, shown in Table 1, was calculated after 24 h using Equation (2). As can be seen, PIM 1, which did not contain any plasticizer, was not able to transport Cd. However, from Figure 2a, where the transient concentration curves are presented for this PIM, it is observed that even though the extraction of the metal occurred (depletion of the feed phase), it was not transported to the stripping phase. In comparison, in the case of PIM 2 (see Figure 2b), the metal extracted in the feed phase was released in the stripping compartment, reaching a transport efficiency of 82.2%. This good performance was observed for all the PIMs containing a plasticizer—independently of their nature (NPOE and FNPOE are aromatic compounds while DBS is aliphatic) and their characteristics (high or low dielectric constant, for example). The need to add a plasticizer to enable the transport of Cd in a PIM was also observed using Aliquat 336 as the carrier [18].

The transport process can be described by the reaction between the metal and the carrier (R_4_P^+^Cl^−^) present in the PIM:

*n* R_4_P^+^Cl^−^_PIM_ + [CdCl*_n_*_+2_]^n−^_(aq)_ → [(PR_4_)*_n_* CdCl*_n_*_+2_] _PIM_ + *n* Cl^−^_(aq)_ (3)

where n can be one or two, and the subscripts aq and PIM denote the aqueous and membrane phases, respectively. It is worth mentioning that when Aliquat 336 is used as the extractant, it has been reported that CdCl_4_^2−^ species are responsible for the extraction in non-viscous media [3], whereas CdCl_3_^−^ species are responsible for the extraction when viscous diluents are used [18].

The stripping step is understood to be the release of Cd in ultra-pure water due to the fact that the predominant species of the metal in this medium (Cd^2+^) cannot interact with the carrier. Moreover, other stripping solutions were tested using PIM 2, such as a 0.5 M HNO_3_ or a 0.1 M EDTA solution. It was found that the transport of Cd using nitric acid followed the same trend as ultra-pure water. In this case, stripping is due to both the predominance of Cd^2+^ and the interaction of the nitrate anion with (R_4_P)^+^, which forms the (R_4_P)^+^ NO_3_^−^ ion pair [19]. However, EDTA solution only allowed for the recovery of about 20% of the metal and thus this reagent was discarded in further experiments.

### 3.3. Cadmium Preconcentration Using the PIM-Device

**Figure 3:**
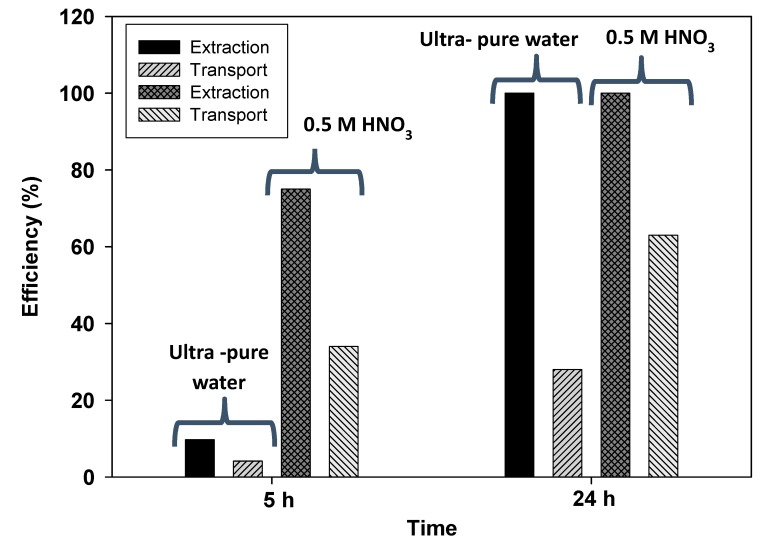
Effect of the stripping phase composition on Cd extraction and recovery vs. time. Feed phase: 0.5 mg L^−^^1^ Cd in 0.5 M NaCl. PIM 2.

Based on the transport results, it was decided that a PIM 2 was to be used in the device and to investigate its capacity for the preconcentration of very low Cd content solutions in 0.5 M NaCl (to resemble a seawater sample), which also tested nitric acid and ultra-pure water as stripping solutions. The results, presented in Figure 3, show that in both cases it was necessary to lengthen the experiment to 24 h to obtain quantitative extraction and a 0.5 M HNO_3_ allowed the highest transport value. However, it should be noted that in this more favorable case, 37% of the initial metal remained in the PIM. The fact that the extracted metal is not completely released in the stripping phase, as occurred when using the two-compartment transport cell, may be due to the lack of agitation in this latter phase.

Given this, in order to enhance transport and release in the stripping phase, we tested the composition of other membranes with a higher amount of plasticizer, 40% instead of 20% (for both NPOE and DBS), while maintaining the same amount of CTA (50%) with the aim of decreasing the viscosity of the PIM. By means of these new PIMs, and using a 0.5 M HNO_3_ as the stripping phase, it was found that all the Cd extracted in the feed phase was quantitatively transported to the stripping compartment after 24 h for the PIM with NPOE, while the PIM with DBS transported 93% of the initial metal. Thus, membranes with only 10% of the carrier in addition to a lower viscosity, allowed for both effective extraction and transport, which solved the problem that the lack of agitation of the small volume of stripping phase posed. Finally, a PIM consisting of 50% CTA + 10% THTDPCl + 40% NPOE, incorporated in the device, was used for the preconcentration of 10 μg L^−^^1^ of Cd in both 0.5 M NaCl and spiked seawater samples. The results are presented in Table 2 where it can be seen that the developed preconcentration method allows for the total recovery of the metal in the stripping phase. The fact that the values are similar in both solutions shows that Cd transport is not affected by other ions present in seawater. Moreover, since the metal is preconcentrated and released in a clean matrix (nitric acid), it facilitates its determination by spectrophotometric techniques, which are not suitable for the direct measurement of the metal in high saline solutions.

## 4. Conclusions

A successful transport of Cd from chloride solutions has been accomplished using a PIM consisting of CTA as the polymer, THTDPCl as the carrier, and DBS or NPOE as the plasticizer. Even though both ultrapure water and nitric acid solutions are suitable stripping solutions, when the PIM is used for preconcentration purposes, a 0.5 M HNO_3_ solution is more efficient. A membrane consisting of 50% CTA + 10% THTDPCl + 40% NPOE enabled Cd transport from seawater samples containing only 10 µg L^−^^1^, achieving in a single step both metal preconcentration and a matrix clean-up. These results show that the PIM system designed here is an efficient analytical tool that should be considered for Cd monitoring in complex natural waters.

## Figures and Tables

**Table 1:** Effect of PIM (polymer inclusion membrane) composition on Cd transport efficiency (24 h) and the characteristics of the plasticizers used to prepare the membranes. Feed phase: 10 mg L^−^^1^. Stripping phase: ultra-pure water.

PIM	Polymer (CTA) (%)	Carrier (THTDPCl) (%)	Plasticizer			TE (%)
			Content (%)	Viscosity (cP)	Dielectric Constant (ε)	
1	70	30	0	-	-	0
2	50	30	NPOE (20)	12.8	23.1	82.2
3		30	FPNPOE (20)	13	50	71.8
4		30	DBS (20)	9.5	4.5	84.1

**Table 2:** Cd preconcentration using the PIM-device (24 h). PIM: 50% CTA + 10% THTDPCl + 40% NPOE.

Sample	Cd Stripping Phase (µg L^−1^)	TE (%)	Recovery (%) ± SD
0.5 M NaCl + 10 µg L^−1^ Cd	238.33	119	108 ± 15
	194.30	98	
Seawater + 10 µg L^−1^ Cd	202.20	101	99 ± 4
	191.43	96	
